# Engineered Mesenchymal Stromal Cells in Oncology: Navigating Between Therapeutic Delivery and Tumor Promotion

**DOI:** 10.3390/genes17010108

**Published:** 2026-01-20

**Authors:** Marta Warzycha, Agnieszka Oleksiuk, Olga Suska, Tomasz Jan Kolanowski, Natalia Rozwadowska

**Affiliations:** 1 Institute of Human Genetics, Polish Academy of Sciences, 60-479 Poznan, Poland; marta.warzycha@famicordtx.com (M.W.); tomasz.kolanowski@famicordtx.com (T.J.K.); 2 FamiCordTX S.A., 02-234 Warsaw, Poland; olga.suska@famicordtx.com; 3 Faculty of Chemistry, University of Warsaw, 02-093 Warsaw, Poland; a.oleksiuk4@student.uw.edu.pl

**Keywords:** MSCs, ex vivo gene therapy, ATMP, cancer, therapeutic agent

## Abstract

Mesenchymal stromal cells (MSCs) are intensively investigated in oncology owing to their intrinsic tumor-homing ability and capacity to deliver therapeutic agents directly into the tumor microenvironment (TME). Recent advances in genetic engineering have enabled precise modification of MSCs, allowing controlled expression of therapeutic genes and other cargo delivery, thus improving targeting efficiency. As cellular carriers, MSCs have been engineered to transport oncolytic viruses, suicide genes in gene-directed enzyme prodrug therapy (GDEPT), multifunctional nanoparticles, and therapeutic factors such as IFN-β or TRAIL, while engineered MSC-derived extracellular vesicles (MSC-EVs) offer a promising cell-free alternative. These strategies increase intratumoral drug concentration, amplify bystander effects, and synergize with standard therapies while reducing systemic toxicity. Conversely, accumulating evidence highlights the tumor-promoting properties of MSCs: once recruited by inflammatory and hypoxic cues, they remodel the tumor microenvironment by stimulating angiogenesis, suppressing immune responses, differentiating into cancer-associated fibroblasts, and promoting epithelial-to-mesenchymal transition (EMT), ultimately enhancing invasion, metastasis, and therapy resistance. This duality has sparked both enthusiasm and concern in the oncology field. The present review outlines the paradoxical role of MSCs in oncology—ranging from their potential to promote tumor growth to their emerging utility as vehicles for targeted drug delivery. By highlighting both therapeutic opportunities and biological risks, we aim to provide a balanced perspective on how MSC-based strategies might be refined, optimized, and safely integrated into future cancer therapies.

## 1. Introduction

Since their first identification in adult bone marrow in 1976 [1], mesenchymal stromal cells (MSCs) have been recognized as a multipotent cell population widely distributed throughout vascularized tissues, typically residing in perivascular niches [2]. For example, MSCs have been identified in skeletal muscle, adipose tissue, dental pulp, tendons, umbilical cords, amniotic fluid, and placenta, among others [1,2]. More recently, alternative sources such as menstrual blood and endometrial tissue have emerged as non-invasive, renewable reservoirs for human MSCs [3]. MSCs are defined by their ability to self-renew and differentiate into multiple mesenchymal lineages. MSCs also secrete a broad range of paracrine factors and can regulate both innate and adaptive immune responses, affecting numerous physiological and pathological processes.

MSCs must meet three minimal criteria established by the International Society for Cellular Therapy (ISCT) in 2006 to be properly identified in vitro. These include adherence to plastic under standard culture conditions, the ability to differentiate into osteoblasts, adipocytes, and chondrocytes, and a specific surface marker profile: expression of CD73, CD90, and CD105 in over 95% of cell population alongside minimal presence of hematopoietic markers (in less than 2% of cell population), such as CD34, CD45, and HLA-DR [2]. Despite their presence in a wide range of niches, MSCs derived from bone marrow, adipose tissues, and extraembryonic tissues remain the most promising for clinical applications. MSCs have a long history of clinical use both in clinical trials and hospital exemption (HE) approaches (a regulatory pathway under European Advanced Therapy Medicinal Products (ATMP) Regulation that permits hospitals to prepare and administer customized, non-routine advanced therapies for individual patients without full market authorization, after meeting defined conditions) [4]. Taken together, the regenerative and immunomodulatory capacities of MSCs make them promising candidates for cell-based therapies. MSCs have been tested in a wide range of conditions involving tissue repair and regeneration, immune modulation, and inflammation mitigation [5]. According to data reported by the US National Institutes of Health (https://clinicaltrials.gov), as of August 2025, around 500 MSC-based clinical trials either have been completed or remain ongoing. One of the most recent studies also concerns oncological diseases. Ramos-Fresnedo et al. presented a clinical trial protocol utilizing allogeneic adipose-derived MSCs for the treatment of patients with recurrent glioblastoma or astrocytoma undergoing surgical resection (NCT05789394) [6].

The ability of MSCs to migrate towards sites of inflammation and injury—including inflamed tumor sites—has raised questions about their potential role in cancer. This process of migration is often referred to as homing, since in this context the tumor site could be defined as “non-healing wound” [7]. Key mechanisms involved in this process include the release plethora of chemoattractants, growth factors, and cytokines by tumor cells. Homing consists of a series of coordinated steps: (a) tethering and rolling facilitated by selectins, (b) activation in response to inflammatory signals, (c) arrest by integrins, (d) transmigration or diapedesis supported by matrix metalloproteinases, and (e) migration driven through a chemokine gradient. It should be noted that homing includes both systemic (MSCs are administered intravenously or recruited endogenously into circulation) and non-systemic homing (MSCs are locally transplanted to the target tissue and then directed to the site of injury by a chemokine gradient) [8]. Moreover, a range of biological, biochemical, and biophysical factors critically regulate MSCs’ survival and homing, mostly through interactions among cells, extracellular matrix, and bioactive molecules in vivo and in vitro [9].

While MSCs derived from different sources must fulfill the previously mentioned criteria, they often display differences in their transcriptomic profiles. It is important to note that MSCs do not constitute a uniform cell population, with biological differences between MSCs from different individuals and inter-individual variation between MSCs of different ages. These factors collectively impact the success of the treatment outcome [8,10,11]. Several initial reports published about 20 years ago suggested that MSCs may have the ability to transform into tumor cells; however, all of those findings were verified to be a result of cross-contamination with transformed cells [12]. These events have led to considerable discussion about the quality of ATMPs prepared by manufacturing sites worldwide and highlight the need for establishing improved standards for further product development.

In the following sections, we examine how genetic modification has redefined the role of MSCs in oncology, transforming them from potential facilitators of tumor growth into promising agents of targeted cancer therapy. Through the introduction of therapeutic genes, prodrug-converting enzymes, or oncolytic factors, engineered MSCs can amplify their natural tumor-homing capacity and immunomodulatory functions to achieve selective antitumor effects. Nevertheless, this therapeutic potential must be viewed in light of the inherent duality of MSCs. Unmodified MSCs can promote tumor progression by supporting angiogenesis, suppressing immune responses, and facilitating metastasis, whereas these same biological properties—migration toward tumor sites, secretion of bioactive molecules, and capacity for microenvironmental interaction can be harnessed for therapeutic purposes. By exploring both aspects of this paradox, we aim to synthesize current understanding of MSCs’ multifaceted functions in cancer and to assess how genetic engineering can optimize their use in next-generation oncologic therapies.

## 2. MSCs as Therapeutic Tools in Cancer Treatment

MSCs are actively being explored as platforms for the targeted delivery of anticancer agents. Their ability to migrate towards tumor-associated signals has been harnessed in various strategies, where MSCs serve as cellular vectors to transport biologically active molecules directly into the tumor microenvironment. In recent years, MSCs have been engineered or combined with complementary technologies—such as oncolytic viruses, nanoparticles, exosomes, proteins, and therapeutic genes—to enhance the precision, potency, and safety of cancer treatments.

### 2.1. Oncolytic Viruses

Oncolytic viruses (OVs) are genetically engineered or naturally occurring viruses that selectively replicate within and destroy tumor cells, while leaving healthy tissues largely unaffected. Beyond direct oncolysis, they trigger potent antitumor immune responses by releasing tumor-associated antigens and engaging both innate and adaptive immunity, which contributes to durable systemic protection against cancer [13,14]. The clinical potential of OVs was highlighted in 2015, when talimogene laherparepvec (T-VEC) became the first oncolytic virus to receive FDA approval for the treatment of melanoma, marking a major milestone in the development of virotherapy [15]. Since then, a variety of viruses—including adenoviruses, herpes simplex viruses, vaccinia viruses, reoviruses, and measles viruses—have been engineered and tested as therapeutic agents in oncology [16]. Despite encouraging clinical outcomes, their systemic delivery remains a major obstacle. Intravenous administration often results in rapid viral clearance by pre-existing antiviral immunity, and only a small fraction of OVs successfully reach tumor sites when direct intratumoral injection is not feasible. This limitation has led to the exploration of MSCs as natural carriers of OVs. MSCs can protect viral particles from immune neutralization in circulation, exploit their intrinsic tumor-homing capacity to deliver OVs more efficiently, and thereby enhance the therapeutic efficacy of virotherapy [13].

As an illustration, studies in gliomas—highly aggressive and therapy-resistant malignancies—highlight the promising efficacy of oncolytic virus–based MSC therapies. Preclinical studies demonstrated that MSCs loaded with oncolytic adenoviruses could migrate toward invasive glioma regions, protect viral particles from immune clearance, and amplify local viral replication within the tumor mass. These findings have provided the rationale for clinical translation [17,18]. As an example, Ning and colleagues conducted a Phase I trial (NCT05717699) in patients with recurrent high-grade gliomas using an oncolytic adenovirus expressing non-secreting human interleukin-12 (Ad-TD-nsIL-12). Ad-TD-nsIL12 could be administered repeatedly without safety concerns, and this therapy has the potential to control tumor growth and achieve therapeutic efficacy; however, this study has limitations due to the small sample size [17]. Similarly, Lang et al. tested adenovirus-mediated p53 gene therapy in recurrent gliomas, reporting acceptable safety and biological activity within tumors, though clinical responses were modest [18]. These early-phase studies highlight both the therapeutic potential and the limitations of OV-based strategies in glioma, emphasizing the need for improved delivery systems [17,18]. Building on these findings, MSCs could potentially serve as cellular carriers for adeno-associated viral (AAV) vectors, enabling localized delivery of therapeutic genes while minimizing systemic exposure. Such an MSC-AAV strategy may represent a next-generation approach for cancer therapy, potentially advancing into early-phase clinical evaluation. In this regard, MSCs are being explored as promising cellular carriers to enhance OV targeting, reduce off-target effects, and ultimately increase the efficacy of virotherapy in difficult-to-treat brain tumors.

### 2.2. Gene-Directed Enzyme Prodrug Therapy (GDEPT)

Gene-directed enzyme prodrug therapy (GDEPT) is a cancer treatment strategy developed to overcome the limitations of conventional chemotherapy, such as systemic toxicity and lack of tumor specificity [19]. The central principle of GDEPT is the separation of drug delivery from drug activation. Instead of administering a cytotoxic compound directly, a non-toxic prodrug is given systemically. Within the tumor, this prodrug is converted into its active, toxic form by an enzyme encoded by an introduced therapeutic gene. This approach allows high concentrations of the active compound to be generated directly at the tumor site, while minimizing exposure of healthy tissues. At its core, GDEPT consists of three essential components: a non-toxic prodrug that can circulate safely through the body, a suicide gene encoding an enzyme capable of metabolizing that prodrug into a cytotoxic compound, and a vector delivering the suicide gene into tumor cells. Once transduction occurs, the enzyme expressed in tumor tissue converts the inactive compound into its active form, producing a localized chemotherapeutic effect. Importantly, this process also generates a bystander effect, whereby adjacent, non-transduced tumor cells are killed due to the diffusion of toxic metabolites or intercellular communication, amplifying the overall therapeutic impact [19,20,21]. In the literature, this strategy appears under several different names, including virus-directed enzyme prodrug therapy, suicide gene therapy, and gene-activated prodrug therapy [20,21]. While the terminology varies, the underlying concept remains the same: introducing an enzyme-encoding gene that locally converts a non-toxic prodrug into a cytotoxic agent within tumor tissue. Despite its promise, one of the major challenges of GDEPT is the efficient and tumor-specific delivery of suicide genes, since viral and non-viral vectors often face biological barriers and safety concerns. To address this issue, researchers have investigated the use of MSCs as genetic cargo carriers. Leveraging their tumor-homing ability, MSCs can transport therapeutic genes directly into the tumor microenvironment, thereby increasing the specificity and effectiveness of GDEPT-based cancer therapies.

Enzymes used in GDEPT can be broadly divided into two categories. The first group is composed of enzymes of human origin, such as cytochrome P450, which generally elicit a weaker immune response but may carry a risk of toxicity in non-target tissues. The second group is derived from other sources, including viral thymidine kinase (TK), cytosine deaminase (CD) from bacteria or yeast, purine nucleoside phosphorylase (PNP), and bacterial nitroreductase (NTR). While these enzymes can trigger stronger immune reactions, they are less likely to cause off-target toxicity, making them attractive candidates for therapeutic applications [22].

Several enzyme–prodrug systems have been successfully tested in combination with MSCs, providing proof-of-concept for this strategy. One of the most extensively studied is the cytosine deaminase/5-fluorocytosine (CD/5-FC) system, in which the microbial enzyme converts the non-toxic 5-FC into 5-fluorouracil (5-FU), a metabolite that interferes with DNA and RNA synthesis, leading to cytotoxicity and cell death [23]. A compelling illustration of the therapeutic promise of MSC-mediated GDEPT comes from glioblastoma research. In an orthotopic mouse model of glioma, MSCs engineered with the CD/5-FC system markedly suppressed tumor growth and induced a strong bystander effect, eliminating neighboring cancer cells. Notably, when this approach was combined with temozolomide (TMZ)—the current standard of care for glioblastoma—the two treatments acted synergistically, resulting in enhanced DNA damage, tumor regression, and significantly prolonged survival compared with either therapy alone [24]. Another example is the herpes simplex virus thymidine kinase/ganciclovir (HSV-TK/GCV) system, where HSV-TK phosphorylates ganciclovir into toxic nucleotides that disrupt DNA synthesis and trigger apoptosis, with cytotoxic metabolites spreading to neighboring cells via gap junctions to induce a strong bystander effect. Recent work has demonstrated that MSC from human exfoliated deciduous teeth (SHED), genetically modified to express a less toxic HSV-TK variant (TKA168H), retained high proliferative capacity and exhibited targeted migration towards non-small cell lung cancer (NSCLC) cells in the brain. In a mouse model of NSCLC brain metastases, intratumoral implantation of SHED-TK followed by systemic GCV administration not only suppressed tumor growth but also markedly prolonged survival compared with controls, in some cases completely eradicating tumors [25].

Collectively, these examples illustrate the versatility of GDEPT strategies when combined with MSCs, demonstrating their capacity to achieve localized prodrug activation, induce potent bystander killing, and synergize with conventional treatments. While approaches such as CD/5-FC and HSV-TK/GCV have provided compelling preclinical proof-of-concept across different tumor models, further optimization is required to ensure efficient gene delivery, sustained enzyme expression, and safety in clinical translation. Nevertheless, MSC-based GDEPT remains a promising avenue for developing more precise and effective anticancer therapies.

### 2.3. Nanoparticles

Nanoparticles (NPs) are engineered nanostructures with dimensions typically ranging from 1 to 100 nm in size and are commonly classified into three groups: organic NPs (e.g., micelles, dendrimers, and liposomes/lipid nanoparticles); inorganic NPs (metals such as gold, silver, and iron-oxide; metal oxides such as titanium dioxide, zinc oxide, and silica; semiconductors such as quantum dots); and carbon-based nanomaterials (fullerenes, graphene, graphene oxide, carbon nanotubes, carbon nanofibers, and activated carbon) [26]. The unique physicochemical properties of nanomaterials, such as high surface area, adjustable size, and capacity for functional modification, when combined with MSCs, hold great potential in therapeutic fields by enabling more precise, targeted, and effective treatments [27]. MSCs possess an intrinsic ability to home to tumor sites, while NPs can be used to regulate their controlled and localized release, ensuring that therapeutic effects are concentrated in the pathological tissue. Moreover, their optical and magnetic properties can be exploited to enable real-time, non-invasive monitoring of cell migration, proliferation, and differentiation in the patient’s body through imaging modalities such as MRI [28,29], SPECT [30], or near-infrared (NIR) fluorescence imaging [31].

NPs, such as polymeric micelles and liposomes, are employed to encapsulate chemotherapeutic agents—including paclitaxel, doxorubicin, gemcitabine, and carboplatin—to enhance tumor-specific delivery and minimize off-target toxicity. Zhao et al. presented an approach where doxorubicin-containing polymer NP loaded into MSCs was used in pulmonary melanoma metastases therapy. Both in vitro and in vivo anti-tumor studies demonstrated that drug-loaded MSCs exhibited therapeutic efficacy against the tumor model [32]. Evidence from a different study conducted by Layek et al. proved that nano-engineered MSCs (with poly(lactide–co–glycolide, PLGA) carrying paclitaxel can significantly improve the anti-cancer efficacy of conventional chemotherapeutic drugs on an orthotopic lung tumor model with significantly less off-target deposition [33]. A similar approach was used by Wang et al., where paclitaxel encapsulated with PLGA NPs was used for orthotopic glioma rats and demonstrated enhanced tumor targeting and increased survival in orthotopic glioma–bearing rats following contralateral injection [34]. According to a separate study published in 2025, researchers used magnetic nanoparticles (MNPs) functionalized with disulfide bonds to co-deliver gemcitabine and a miRNA34a mimic targeting pancreatic cancer cells, enhancing gemcitabine sensitivity, and lowering toxicity, which was presented on a model of healthy keratinocytes [35].

It should be noted that both the preparation process and the physicochemical characteristics of nanoparticles can significantly impact the biological behavior of MSCs. The nature of the nanoparticles may influence cell viability and modify the pharmacokinetic and pharmacodynamic profiles of the encapsulated drug. Furthermore, the application of MSCs as vectors for nanoparticle-mediated drug delivery may result in inefficient tumor localization, elevated clearance by reticuloendothelial organs, and toxicity concerns [36]. Comprehensive assessment of the biophysical characteristics and corresponding biological effects of nanomaterials is therefore required to identify those that maintain MSC viability and membrane integrity. Future research should focus on developing nanoparticles with higher internalization capacity; for example, the covalent attachment or physical adsorption of nanoparticles onto the MSC surface can substantially improve drug-loading efficiency and promote endocytic uptake [37].

These findings highlight the safety and feasibility of integrating NPs with MSC-based therapies, supporting their dual role as imaging agents and therapeutic delivery vehicles in oncology.

### 2.4. Ex Vivo Gene Therapy and Therapeutic Proteins Delivery

The inherent ability of mesenchymal stromal cells (MSCs) to migrate towards tumor sites and integrate into the tumor microenvironment has been increasingly exploited in ex vivo gene therapy approaches. In this strategy, MSCs are genetically modified outside the patient’s body to express therapeutic transgenes and subsequently administered as living drug factories capable of producing and releasing bioactive molecules directly within tumors. Through such genetic modification, MSCs can be engineered to secrete cytokines and other biologically active molecules directly within tumors, allowing sustained and site-specific release while minimizing systemic toxicity. This approach enables higher local concentrations of therapeutic agents and enhances antitumor efficacy, particularly in solid tumors that are otherwise difficult to target through conventional systemic administration.

Among the proteins explored for MSC-based delivery, interferon-β (IFN-β) has received considerable attention due to its potent antiproliferative, antiangiogenic, and immunomodulatory properties [38]. Studeny and colleagues first demonstrated that bone marrow-derived MSCs engineered to secrete human IFN-β could home to melanoma xenografts, where they significantly inhibited tumor growth and prolonged survival. This antitumor effect was attributed primarily to the direct antiproliferative action of human IFN-β on malignant cells, since systemic administration of recombinant IFN-β or MSCs secreting IFN-β at distant sites failed to reproduce these benefits [39]. Subsequent work by Kidd et al. extended these findings to pancreatic cancer, showing that MSCs expressing IFN-β localized to both primary and metastatic lesions, leading to a marked suppression of tumor burden. Interestingly, this effect was dependent on the inflammatory tumor microenvironment; treatment with the potent anti-inflammatory agent CDDO-Me reduced MSC engraftment and diminished the efficacy of IFN-β delivery [40].

Another prominent strategy involves engineering MSCs to secrete tumor necrosis factor-related apoptosis-inducing ligand (TRAIL), a pro-apoptotic cytokine that selectively induces apoptosis in malignant cells by engaging death receptors while sparing most normal tissues [41,42]. Preclinical studies have consistently shown the therapeutic potential of MSC-delivered TRAIL across various cancer models. For instance, Luetzkendorf and colleagues demonstrated that lentivirally engineered TRAIL-MSCs inhibited colorectal carcinoma (CRC) growth in vitro and in vivo, including in cell lines otherwise resistant to soluble TRAIL. Notably, the therapeutic effect required substantial intratumoral engraftment of TRAIL-MSCs, underscoring the importance of efficient tumor integration for sustained local activity [41]. Similarly, Loebinger et al. showed that systemically administered TRAIL-MSCs homed to lung metastases and, upon induction, mediated complete eradication of metastatic disease in up to 38% of mice in a breast cancer model, while also reducing tumor burden in subcutaneous xenografts. Importantly, these studies reported no systemic toxicity, highlighting the tumor specificity of this approach [42].

These findings underscore the therapeutic potential of MSCs as delivery systems for antitumor proteins. By enabling sustained, localized cytokine release, MSC-based protein therapies can overcome the limitations of conventional recombinant protein administration—such as short half-life, poor bioavailability, and systemic toxicity—and represent a promising direction for the development of more precise and effective cancer treatments.

### 2.5. Engineered MSC-EVs

Mesenchymal stromal cell-derived extracellular vesicles (MSC-EVs) have recently emerged as a promising alternative to whole-cell therapies. EVs are nanosized, membrane-bound vesicles secreted by virtually all cell types, mediating intercellular communication by transferring proteins, nucleic acids, and lipids. They are generally classified into three broad categories: exosomes (30–150 nm), microvesicles (100–1000 nm), and apoptotic bodies (100–5000 nm), which differ in size and biogenesis [43].

MSC-EVs have gained significant attention as a cell-free therapeutic approach, as they recapitulate many of the regenerative and immunomodulatory properties of their parental MSCs, while avoiding key risks associated with cell transplantation, such as immune rejection or malignant transformation [43,44]. Through engineering strategies such as preconditioning, surface modification, or drug/nucleic acid loading, MSC-EVs can be tailored to enhance their therapeutic efficacy in cancer, regenerative medicine, and neurological disorders. Recent evidence highlights the therapeutic potential of MSC-EVs in oncology through their ability to transport a wide spectrum of bioactive molecules. Among these cargos, microRNAs (miRNAs) are of particular interest due to their central role in regulating gene expression. Aberrant expression of miRNAs has been closely associated with cancer initiation and progression, as dysregulated levels can either enhance oncogene activity or silence tumor suppressor genes, thereby promoting uncontrolled proliferation, apoptosis resistance, and metabolic reprogramming [45]. Restoring physiological miRNA profiles thus represents a promising therapeutic strategy. A notable example is exosomal miR-499a-5p, which was found to be significantly downregulated in endometrial cancer. When delivered via MSC-derived exosomes, miR-499a-5p suppressed cancer cell proliferation and angiogenesis both in vitro and in vivo, ultimately inhibiting tumor growth and metastasis by directly targeting VAV3 [46]. These findings underscore the potential of engineered MSC-EVs as vehicles for therapeutic miRNA delivery, offering a targeted approach for cancer treatment. In another report, MSC-EVs were used as a method of delivery aforementioned TRAIL cytokine, where MSC-EVs expressing TRAIL were highly efficient at selectively inducing apoptosis in cancer cells, moreover with partially overcoming TRAIL resistance in cancer cells [47]. MSC-EVs themselves, without payload, have been recognized as a pro-apoptotic agent on Lewis lung carcinoma (LLC), but without significant effect on CT26, B16F10, and TC1 cell lines. Additionally, in this study, MSC-EV were developed using Renilla luciferase (Rluc) for bioluminescent imaging [48]. In an analogous study that employed only MSC-derived EV, it was demonstrated that MSC-EV could significantly inhibit proliferation, migration, and colony formation of cultured 4T1 murine breast cancer cells but without inducing apoptosis [49]. MSC-derived EV can be used as a carrier for chemotherapeutic agents, including paclitaxel. Pascucci et al. reported for the first time that MSCs are able to package and deliver active drugs through their vesicles [50].

Despite the emerging potential of MSC-EVs as biological delivery vehicles, additional technological and engineering refinements are essential to increase drug-loading efficiency, improve targeting accuracy, and strengthen safety profiles. Subsequent studies are also required to generate direct evidence for their clinical translation [51]. Moreover, challenges of MSC-EV production have to be considered (their isolation, characterization, and large-scale manufacturing are complex and expensive processes) [52,53].

It is important to highlight that reporter molecules can be loaded into MSCs and potentially used to facilitate imaging and tracking of tumor progression. For instance, in MDA-MB-231 breast and CAL62 thyroid models, bioluminescent imaging visualized MSC accumulation at tumors and metastases, with ex vivo confirmation [54].

**Figure 1:**
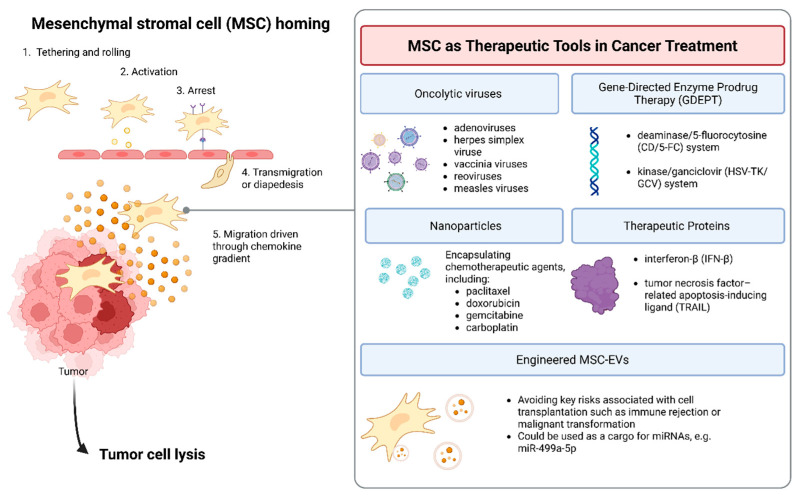
Mesenchymal stromal cell (MSC) homing and therapeutic applications in oncology. MSCs exhibit intrinsic tumor-homing capacity, enabling their use as cellular vehicles for targeted cancer therapy. The left panel illustrates the multistep process of MSC homing to tumor tissue: (1) tethering and rolling on activated endothelium, (2) activation by inflammatory cytokines, (3) arrest, (4) transmigration or diapedesis across the endothelial barrier, and (5) chemokine-driven migration toward the tumor microenvironment, where MSCs contribute to tumor cell lysis. The right panel summarizes key therapeutic strategies exploiting genetically modified MSCs. Engineered MSCs can deliver oncolytic viruses (e.g., adenovirus, HSV, vaccinia, reovirus, measles virus), mediate gene-directed enzyme prodrug therapy (GDEPT) via CD/5-FC or HSV-TK/GCV systems, or serve as carriers for nanoparticles encapsulating chemotherapeutic agents (paclitaxel, doxorubicin, gemcitabine, carboplatin). They may also secrete therapeutic proteins such as interferon-β (IFN-β) or TRAIL. MSC-derived extracellular vesicles (MSC-EVs) represent a cell-free alternative, reducing risks associated with cell transplantation while enabling delivery of regulatory RNAs (e.g., miR-499a-5p). Created in BioRender, https://BioRender.com/uqmin78 (accessed on 15 November 2025).

Below, we provide a comprehensive overview of the strategies in which MSCs are utilized in oncology, as depicted in Table 1 and Figure 1.

## 3. Potential Risks and Tumor-Promoting Effects of MSC-Based Therapies

As stated above, MSCs are actively recruited to tumor sites by chronic inflammatory stimuli, where they integrate into the tumor stroma and modulate tumor growth through direct cell–cell interaction, secretion of paracrine mediators, and by transferring bioactive molecules via extracellular vesicles (EVs). It should be emphasized that MSC homing efficiency is highly variable, generally ranging from 1 to 30% within days after administration, and is influenced by dose, route of delivery, and engineering. In xenografts, basal homing is often <5% but can increase to 20–40% with CXCR4 overexpression or nanoparticle modification. Models with strong chemokine gradients, such as pancreatic cancer and glioblastoma, reach 25–30%, whereas prostate and sarcoma models typically fall below 10% unless enhanced. The source of MSCs also significantly influences tumor homing; for example, bone marrow-derived MSCs display pronounced homing to solid tumors, achieving 10–30% efficiency, which is attributed to high CXCR4 expression, while adipose-tissue-derived MSCs preferentially migrate to breast cancer and melanoma (15–25%) but are less effective in glioblastoma [55,56].

Across various tumor types, MSCs can contribute to cancer hallmarks, including increased proliferation, drug resistance, epithelial to mesenchymal transition (EMT), invasion and metastasis, angiogenesis, and apoptosis suppression [57]. Hypoxic conditions within the tumor microenvironment (TME) further boost MSC recruitment by increasing receptor expression and stimulating the release of factors like vascular endothelial growth factor (VEGF) [58]. Upon arrival at the tumor site, interaction with local cytokines and growth factors induces phenotypic changes in MSCs, shifting their role from tissue regeneration towards tumor growth support [59]. Evidence indicates that MSCs play a role in tumor growth and progression in diverse malignancies, such as prostate [60], colon [61], gastric [62], breast [63], and glioma [64].

It is important to underline that differences based on the tissue source of MSCs (i.e., bone marrow vs. cord blood vs. adipose tissue) cause differences in MSCs’ proliferative capacity, differentiation patterns, and profile of secreted cytokines even across colonies that arise from the same original batch. Differences in individuals from whom MSCs are derived—such as age and health conditions—add to the complexity and heterogeneity of MSCs [10]. For example, umbilical cord-derived MSCs produce and release higher amounts of TGF-β (transforming growth factor-β) and lower levels of VEGF and EGF (epidermal growth factor) than adipose tissue-derived MSCs and amnion-originating MSC [57]. These characteristics influence MSC’s cell phenotype, and their impact on cancer progression remains unpredictable.

### 3.1. Pro-Angiogenic Activity

One major mechanism by which MSCs promote tumor progression is by supporting angiogenesis—the formation of new blood vessels—which is essential for tumor growth and metastasis. Angiogenesis is largely promoted by MSCs through their paracrine activity, involving the secretion of diverse growth factors, cytokines, and EVs that stimulate the development of new vasculature. Significant angiogenic mediators released by MSCs include vascular endothelial growth factor (VEGF, particularly VEGF-C and VEGF-D), basic fibroblast growth factor (bFGF), transforming growth factor-β (TGF-β), platelet-derived growth factor (PDGF), hepatocyte growth factor (HGF), interleukin-6 (IL-6), monocyte chemoattractant protein-1 (MCP-1), and stromal cell-derived factor-1 (SDF-1). These molecules act by enhancing endothelial cell proliferation, migration, and tube formation, while also activating signaling pathways such as MAPK, PI3K/Akt, and Src [65]. These factors stimulate the proliferation and migration of endothelial and smooth muscle cells, ultimately leading to neovascularization within the tumor [66].

Du et al. reported heterogeneous pro-angiogenic properties of MSCs derived from different tissue sources and further demonstrated that their functional heterogeneity is influenced by the in vivo microenvironment rather than by their developmental status. Their study suggests that bone marrow-derived MSCs and placental-derived MSCs may be more suitable for clinical applications in vascular diseases due to their strong paracrine activity [67]. Moreover, MSCs may differentiate into endothelial-like cells or pericytes supporting the vascular structure in tumors. MSCs can exhibit endothelial (CD31, VE-cadherin) or pericyte-like (CD146) phenotypes under defined in vitro conditions, with factors such as VEGF or hypoxia, but their contribution to in vivo outcomes is minimal due to limited MSC engraftment. Human-relevant systems, including xenografts combining adipose tissue-derived MSCs with endothelial colony-forming cells, highlight that functional vessel formation depends largely on paracrine interactions, although hypoxic preconditioning can enhance both paracrine output and differentiation potential. Despite some overlap between mechanisms, paracrine effects remain the dominant contributor to MSC-mediated vascular benefits [68]. TGF-β upregulation correlates with poor prognosis, increased tumor growth, and angiogenesis in several cancers. Conversely, TGF-β signaling antagonists reduce tumor growth and metastasis. Battle et al. demonstrated that under the influence of TGF-β, MSCs can acquire endothelial characteristics contributing directly to the tumor vasculature through p38α signaling, which regulates tumor growth and angiogenesis in both colon cancer xenografts and mouse models [69].

In specific cancer models such as hepatocellular carcinoma [70] and gastric cancer [71], MSCs have been shown to increase microvessel density and promote tumor progression through their angiogenic activities. Cao et al. demonstrated that MSC-derived exosomes have been reported to contain specific pro-angiogenic microRNAs (e.g., miR-21-5p, miR-193a-3p) that can target angiogenesis-related signaling pathways in endothelial cells, ultimately leading to enhanced tube formation and vascular network development [72]. Notably, EVs derived from MSCs generally exhibit pro-angiogenic effects in tumors, although some studies indicate context-dependent roles, with potential anti-angiogenic activity under specific conditions [73,74].

Altogether, MSCs drive tumor angiogenesis by secreting a range of growth factors and cytokines, modulating critical signaling pathways, and potentially undergoing transdifferentiation, all of which synergistically enhance tumor vascularization.

### 3.2. Immunomodulation

MSCs have been extensively characterized as potent immunomodulators capable of influencing both innate and adaptive immune responses. Growing evidence demonstrates their ability to suppress inflammatory processes through the inhibition of immune cell proliferation, differentiation, and effector functions, thereby contributing to the establishment of an anti-inflammatory and tolerogenic microenvironment [75]. MSCs mediate immunomodulation in tumors via soluble factors and direct cell–cell interactions, and these effects are dynamically shaped by the cytokine environment within the TME. Specifically, MSCs can contribute to tumor immune evasion through their immunosuppressive properties. They release various soluble mediators—such as TGF-β, IL-10, prostaglandin E2 (PGE2), and indoleamine 2,3-dioxygenase (IDO)—that inhibit immune cell activation and promote regulatory T cell differentiation, thereby suppressing anti-tumor immune responses. This immunosuppressive milieu helps tumor cells escape immune surveillance, facilitating tumor growth and metastasis [76,77]. In this context, Ling et al. developed a model of murine humanized MSCs capable of expressing IDO (MSCs-IDO). It was determined that through the regulation of the immune response, MSCs-IDO favors the development of melanoma and lymphoma in vivo. Melanoma tumors are characterized by a reduction in the infiltration of CD8+ T-lymphocytes, although no differences are observed in CD4+ T lymphocyte, natural killer (NK) cells, or B lymphocyte infiltration [78]. Studies have shown that MSCs possess immunosuppressive capabilities through EVs. These vesicles regulate immune responses by inhibiting the proliferation of B and T lymphocytes, preventing monocyte differentiation and dendritic cell maturation, while also promoting the generation of regulatory T and B cells as well as M2 macrophages. This immune modulation contributes to the establishment of a tumor-favorable microenvironment, weakening anti-tumor immune responses and enabling cancer cells to escape immune surveillance [79]. Moreover, MSCs have been shown to exert immunosuppressive effects on both T-lymphocytes and NK cells. In an in vitro co-culture model involving bone marrow MSCs, peripheral blood mononuclear cells, and breast cancer cells, bone marrow-derived MSCs were shown to shield tumor cells from immune system-mediated elimination by suppressing the activity of NK cells and cytotoxic T lymphocytes. Moreover, bone marrow-derived MSCs can promote the differentiation of regulatory T cells. This immunomodulatory effect is associated with an increase in Th2-type cytokines and TGF-β, along with a reduction in Th1-type cytokines [80].

In summary, immune evasion within the TME constitutes a pivotal mechanism that enables cancer cells to grow. Among the various factors contributing to this process, MSCs play a central role by modulating the immune landscape through the secretion of a diverse array of immunoregulatory molecules, including cytokines, chemokines, and growth factors. These MSC-derived mediators suppress the cytotoxic activity of T lymphocytes and NK cells, promote the expansion of regulatory T cells, and shift the cytokine milieu toward an anti-inflammatory, tumor-promoting profile. Collectively, these interactions create an immunosuppressive microenvironment that facilitates tumor cell survival, immune escape, and subsequent disease progression.

### 3.3. Differentiation into Cancer-Associated Fibroblasts (CAFs)

Cancer-associated fibroblasts (CAFs) have gained increasing recognition as the critical role of the tumor stroma in cancer progression has become more documented. CAFs’ presence has been consistently linked to poor patient prognosis (circulating CAFs) and is implicated in a wide range of pro-tumorigenic functions that contribute to malignant progression [76]. MSCs can be found among CAFs in the TME and contribute to tumor progression by supporting cancer cell survival, proliferation, and chemoresistance [81].

Another mechanism by which MSCs support tumor progression is through their conversion into CAFs. Upon exposure to tumor-derived signals, MSCs can adopt a CAF-like phenotype, characterized by the expression of markers such as α-smooth muscle actin (α-SMA), fibroblast activation protein (FAP), and PDGFR-β. These MSC-derived CAFs contribute to extracellular matrix remodeling, enhance tumor cell invasion, and secrete a range of pro-tumorigenic factors, including TGF-β, IL-6, and CXCL12. Their presence also supports immunosuppression and promotes angiogenesis, further reinforcing a microenvironment favorable to cancer growth and metastasis [59]. Miyazaki et al. have presented that adipose tissue-derived MSCs can differentiate into distinct CAF subtypes depending on the different co-culture conditions in vitro. Adipose tissue-derived MSCs differentiate into two distinct CAF subpopulations; direct contact co-culture with PDAC (Pancreatic Ductal Adenocarcinoma) cell line Capan-1 induced differentiation into myoblastic CAF (myCAF) and inflammatory CAF (iCAF), while indirect co-culture induced differentiation exclusively into iCAFs. It should be noted that each subtype has a distinct role; myCAF is a CAF subpopulation with an elevated expression of αSMA that is located adjacent to cancer cells, whereas iCAFs are located further away from cancer cells and could be characterized by the secretion of inflammatory mediators such as IL-6 [82]. Pan et al. observed that MSCs with CAF-like phenotype could be a key factor promoting the growth and invasion of B-cell acute lymphoblastic leukemia (B-ALL) cells, and the SDF-1/CXCR4 axis might be a significant factor in mediating the communication of MSCs with CAF-like phenotype and leukemia cells [83]. Compared with MSCs, CAFs typically exhibit high proliferative capacity and secrete elevated levels of VEGF and other immunosuppressive mediators [57]. Particular attention should be drawn to the fact that evidence for MSC-to-CAF transition is currently strongest in preclinical models, including in vitro co-culture systems and mouse xenografts. Direct demonstration in human tumor samples via lineage tracing remains limited due to technical challenges; most human data rely on marker co-expression and functional assays rather than genetic lineage tracing as performed in mouse models. For example, current evidence largely depends on indirect approaches, such as co-expression of CAF markers (α-SMA and FAP) with MSC-associated markers (including PDGFR-β and THY1) and immunohistochemistry analyses in gastric, prostate, bladder, and colorectal cancers, indicating that bone marrow MSCs contribute to the stromal CAF population [84]. Regarding the stability of the CAF-like state derived from MSCs, available data suggest that the CAF-like state is generally maintained under continuous tumor microenvironmental signals, although some plasticity exists, allowing context-dependent functional changes [85].

### 3.4. Promotion of Metastasis via EMT

Epithelial–mesenchymal transition (EMT) plays a central role in promoting tumor metastasis by endowing epithelial cancer cells with enhanced motility, invasiveness, and resistance to apoptosis. It has been shown that MSCs promote EMT in cancer cells, a key step in tumor invasion and metastasis [86,87]. EMT is a biological process during which epithelial cancer cells acquire a more motile, mesenchymal phenotype, allowing them to detach from the primary tumor mass, invade surrounding tissues, and enter the bloodstream, ultimately contributing to metastatic colonization at distant sites [88]. According to Ribatti et al. (2020), EMT involves the loss of epithelial polarity and cell–cell adhesion through downregulation of epithelial markers such as E-cadherin, occludin, desmoplakin, and cytokeratins, accompanied by upregulation of mesenchymal markers including vimentin, fibronectin, and N-cadherin. This phenotypic shift is orchestrated by transcription factors such as Snail1/2 (Slug), Twist, ZEB1/2, and FOXC2, which repress epithelial gene expression and activate mesenchymal programs. The process is further driven by multiple signaling pathways—most notably TGF-β/Smad, Wnt/β-catenin, Notch, and HIF-1α—and modulated by cytokines such as IL-6, IL-8, TNF-α, HGF, and PDGF, frequently secreted by tumor-associated stromal cells [87].

These changes enable circulating tumor cells to survive in the bloodstream, evade immune elimination, and later undergo mesenchymal–epithelial transition (MET) to establish secondary tumors. Furthermore, cancer cells in a partial or hybrid EMT state retain both adhesive and migratory properties, providing a survival advantage during dissemination and colonization. Thus, EMT not only drives the initiation of metastasis but also supports tumor cell adaptability, immune evasion, and successful colonization at distant sites [86,88].

The study by Chen et al. (2019) demonstrates that MSCs can enhance hepatocellular carcinoma (HCC) growth and metastasis by activating the MAPK/ERK pathway and inducing EMT. The authors identified integrin α5 (ITGA5) as a key mediator of MSC-driven hepatocellular carcinoma cells migration and invasion [89].

According to Mele et al. MSCs can trigger EMT in human colorectal cancer cells through surface-bound TGF-β signaling. The induced EMT enhances cancer cell motility and invasiveness, suggesting that MSCs within the TME may promote colorectal cancer progression and metastasis. Promotion of EMT in cancer cells by MSC required cell-to-cell contact and appeared to be mediated by surface-bound TGF-β expressed on MSC upon cross-talk with tumor cells [90]. The studies discussed here predominantly utilize in vitro systems or immunodeficient animal models, which offer mechanistic insights but lack the complexity of an intact immune system. These limitations underscore the need to cautiously interpret the findings and highlight the importance of further validation in immunocompetent models and clinical settings to fully understand the therapeutic potential and biological relevance.

**Figure 2:**
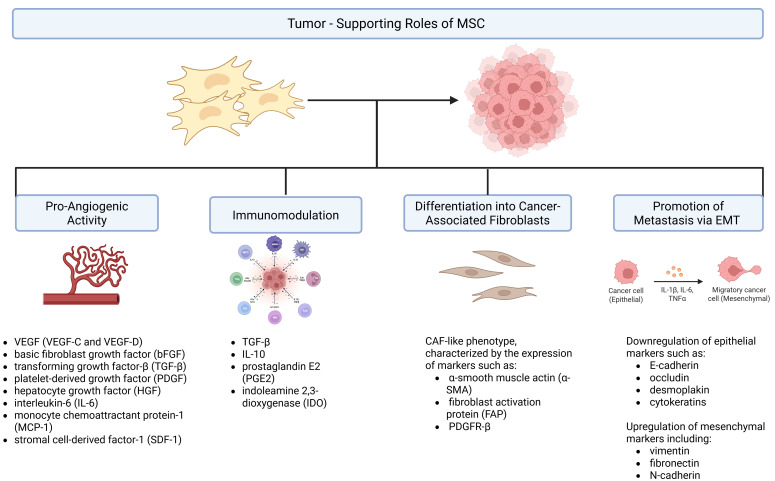
Tumor-supporting roles of mesenchymal stromal cells (MSCs). MSCs can promote tumor progression through multiple mechanisms within the tumor microenvironment. These include pro-angiogenic activity mediated by VEGF family members and other growth factors; immunomodulation driven by cytokines such as TGF-β, IL-10, PGE2, and IDO; differentiation into cancer-associated fibroblasts (CAFs) characterized by α-SMA, FAP, and PDGFR-β expression; and promotion of metastasis via epithelial–mesenchymal transition (EMT), involving downregulation of epithelial markers (e.g., E-cadherin) and upregulation of mesenchymal markers (e.g., vimentin, N-cadherin). Together, these processes support tumor growth, invasion, and dissemination. Created in BioRender, https://BioRender.com/u2lq7qo (accessed on 15 November 2025).

The roles of MSCs in tumor-promoting processes, as described above, are illustrated in Figure 2.

## 4. Conclusions

MSCs play a crucial role in the context of cancer, acting as dynamic components of the tumor microenvironment while simultaneously serving as promising tools for targeted therapy. A deeper understanding of their interactions with cancer cells, stromal elements, and immune regulators is essential to clarify the mechanisms that drive either pro- or anti-tumorigenic effects. Importantly, accumulating evidence suggests that MSC subsets differ in their functional and secretory profiles and that these activities are dynamically shaped by signals from the tumor microenvironment. Exploring these distinctions, as well as identifying factors that govern MSC plasticity, could open new avenues for therapeutic exploitation. Advances in engineering strategies have already positioned MSCs as efficient vectors for delivering genes, proteins, nanoparticles, and extracellular vesicles, offering opportunities for highly specific and personalized treatments. To translate these approaches into clinical reality, future research must focus on resolving MSC heterogeneity, establishing standardized protocols, and integrating MSC-based systems into multimodal oncology regimens. Such efforts hold the potential to transform MSCs from controversial players into reliable allies in the fight against cancer.

## Figures and Tables

**Table 1:** Therapeutic strategies employing engineered MSCs in oncology.

Cargo		MSC Source	Tumor Indication	Model	Main Outcome	Key Reference
Oncolytic Viruses (OVs)	Ad-TD-nsIL12	NA	recurrent high-grade glioma	Phase I clinical trial	Repeated administration was safe; showed signals of tumor growth control	[17]
	Ad-p53	NA	glioma	Phase I clinical trial	minimum toxicity; virus showed biological activity in tumor tissue	[18]
GDEPT	cytosine deaminase/5-fluorocytosine (CD/5-FC)	bone marrow-derived MSCs	glioblastoma	in vivo xenograft	tumor suppression; strong bystander effect; synergy with TMZ	[24]
	herpes simplex virus thymidine kinase/ganciclovir (HSV-TK/GCV)	stem cells from human exfoliated deciduous teeth (SHED)	brain metastasis model of non-small cell lung cancer (NSCLC)	in vivo metastasis model	strong bystander effect; tumor regression; prolonged survival; complete tumor regression in a subset of mice	[25]
Therapeutic Proteins	IFN-β	bone marrow-derived MSCs	melanoma [39], pancreatic cancer [40]	in vivo xenograft	tumor growth inhibition; prolonged survival [39]; tumor suppression [40]	[39,40]
	TRAIL	bone marrow-derived MSCs [41]	colorectal cancer (CRC) [41], lung metastases [42]	in vivo xenograft [41], in vivo metastasis model [42]	apoptosis induction in TRAIL-sensitive and TRAIL-resistant CRC cell lines [41]; metastatic reduction, complete elimination of metastatic disease in 38% of mice; no systemic toxicity [42]	[41,42]
Nanoparticles (NPs)	magnetic nanoparticles (MNPs)	not specified	pancreatic cancer	in vivo xenograft	enhanced sensitivity to gemcitabine, lower toxicity	[35]
Extracellular Vehicles (EVs)	miR-499a-5p	mouse bone-marrow MSCs	endometrial cancer	in vivo xenograft	inhibited tumor growth and angiogenesis; reduced tumor volume, weight, and microvessel density; confirmed direct targeting of VAV3	[46]

## Data Availability

No new data were created or analyzed in this study.

## Abbreviations

The following abbreviations are used in this manuscript:

AAV	Adeno-associated virus
AD-MSCs	Adipose-derived mesenchymal stromal cells
ATMP	Advanced therapy medicinal product
B-ALL	B-cell acute lymphoblastic leukemia
bFGF	Basic fibroblast growth factor
BM-MSC	Bone marrow-derived mesenchymal stromal cell
CAF	Cancer-associated fibroblast
CTC	Circulating tumor cell
CXCL12	C-X-C motif chemokine ligand 12 (stromal cell-derived factor-1, SDF-1)
CXCR4	C-X-C chemokine receptor type 4
EGF	Epidermal growth factor
EMT	Epithelial-to-mesenchymal transition
ERK	Extracellular signal-regulated kinase
EV	Extracellular vesicle
GDEPT	Gene-directed enzyme prodrug therapy
HCC	Hepatocellular carcinoma
HE	Hospital exemption
HGF	Hepatocyte growth factor
HIF-1α	Hypoxia-inducible factor-1 alpha
HLA-DR	Human leukocyte antigen—DR isotype
HSV-TK	Herpes simplex virus thymidine kinase
IDO	Indoleamine 2,3-dioxygenase
IFN-β	Interferon-beta
IL-6	Interleukin-6
IL-8	Interleukin-8
IL-10	Interleukin-10
ITGA5	Integrin alpha-5
LLC	Lewis lung carcinoma
MAPK	Mitogen-activated protein kinase
MCP-1	Monocyte chemoattractant protein-1
MET	Mesenchymal-to-epithelial transition
MNP	Magnetic nanoparticle
MSC	Mesenchymal stromal/stem cell
MSC-EV	Mesenchymal stromal/stem cell-derived extracellular vesicle
NK	Natural killer (cell)
NSCLC	Non-small cell lung cancer
NTR	Nitroreductase
OV / OVs	Oncolytic virus / oncolytic viruses
PDAC	Pancreatic ductal adenocarcinoma
PDGF	Platelet-derived growth factor
PDGFR-β	Platelet-derived growth factor receptor beta
PGE2	Prostaglandin E2
PLGA	Poly(lactide-co-glycolide)
PNP	Purine nucleoside phosphorylase
SDF-1	Stromal cell-derived factor-1 (CXCL12)
TME	Tumor microenvironment
T-VEC	Talimogene laherparepvec
TGF-β	Transforming growth factor-beta
TK	Thymidine kinase
TKA168H	HSV-TK mutant variant TKA168H
TMZ	Temozolomide
TNF-α	Tumor necrosis factor-alpha
TRAIL	Tumor necrosis factor-related apoptosis-inducing ligand
VEGF	Vascular endothelial growth factor
